# Bioactive Metabolites from the Endophytic Fungus *Aspergillus* sp. SPH2

**DOI:** 10.3390/jof7020109

**Published:** 2021-02-02

**Authors:** Viridiana Morales-Sánchez, Carmen E. Díaz, Elena Trujillo, Sonia A. Olmeda, Felix Valcarcel, Rubén Muñoz, María Fe Andrés, Azucena González-Coloma

**Affiliations:** 1 Instituto de Ciencias Agrarias, CSIC, Serrano, 115, 28006 Madrid, Spain; viridianamoralessanchez@gmail.com (V.M.-S.); ruben.mm@ica.csic.es (R.M.); mafay@ica.csic.es (M.F.A.); 2 Instituto de Productos Naturales y Agrobiología, CSIC. Avda. Astrofísico F. Sánchez, 3, 38206 La Laguna, Tenerife, Spain; elena.trujillo@ipna.csic.es; 3 Facultad de Veterinaria, UCM, Av. Puerta de Hierro, s/n, 28040 Madrid, Spain; angeles@vet.ucm.es; 4 Producción Animal, INIA, Av. Puerta de Hierro, 12, 28040 Madrid, Spain; fvalcsan@gmail.com

**Keywords:** endophyte, *Aspergillus ochraceous*, antifungal, neoaspergillic acid, ixodicidal, mellein

## Abstract

In the current study, an ethyl acetate extract from the endophytic fungus *Aspergillus* sp. SPH2 isolated from the stem parts of the endemic plant *Bethencourtia palmensis* was screened for its biocontrol properties against plant pathogens (*Fusarium moniliforme*, *Alternaria alternata,* and *Botrytis cinerea*), insect pests (*Spodoptera littoralis*, *Myzus persicae*, *Rhopalosiphum padi*), plant parasites (*Meloidogyne javanica*), and ticks (*Hyalomma lusitanicum*). SPH2 gave extracts with strong fungicidal and ixodicidal effects at different fermentation times. The bioguided isolation of these extracts gave compounds **1**–**3**. Mellein (**1**) showed strong ixodicidal effects and was also fungicidal. This is the first report on the ixodicidal effects of **1**. Neoaspergillic acid (**2**) showed potent antifungal effects. Compound **2** appeared during the exponential phase of the fungal growth while neohydroxyaspergillic acid (**3**) appeared during the stationary phase, suggesting that **2** is the biosynthetic precursor of **3**. The mycotoxin ochratoxin A was not detected under the fermentation conditions used in this work. Therefore, SPH2 could be a potential biotechnological tool for the production of ixodicidal extracts rich in mellein.

## 1. Introduction

Endophytes are a particularly interesting group of microorganisms that can be isolated from asymptomatic plant tissue. Among fungal endophytes, most species belong to the ascomycota and deuteromycota phyla and might be the producers of several groups of new, unique secondary metabolites [1].

The specific relationship between the host plant and its endophytes includes complex biochemical interactions [2,3]. Endophytes have adapted themselves to their special microenvironments by genetic variation, including uptake of some plant DNA into their own genomes [4]. After long-term coexistence with their host, endophytes can synthesize biologically active substances similar to the secondary metabolites produced by host plants [2,5,6]. The potential of fungal endophytes for producing novel biologically active compounds with promising medicinal or agricultural applications has been demonstrated [7,8]. These molecules can play an important role in communication between organisms, in plant protection, and plant adaptation to habitat and environmental changes [8].

Little is known regarding endophyte biogeography and community assembly. An important role for biome and host phylogeny has been shown for bioactivity (against Chagas and malaria) of endophytic fungi from tropical angiosperms and ferns, showing a higher degree of bioactivity in taxa sourced from cloud forests compared to lowland tropical forests [9]. In this context, targeting plants with unique traits, such as the production of unusual secondary metabolites or found in restricted habitats, has been suggested [10].

The Macaronesian region consists of the Azores and Madeira (Portugal) and the Canary Islands (Spain). The three archipelagos share a volcanic origin, a contrasting landscape, and a gentle climate. These features have created an ideal environment for particularly rich biodiversity. The Canaries host unique forests habitats home to the richest biodiversity of the region, and one of the richest in the world. Around 25% of the flora species are endemic [11] and, therefore, hosts of a potentially rich endophytic microbiome.

Endophytes isolated from endemic species of the Laurel forest of the Canarian flora reported as being insecticidal, produced extracts with crop protection effects such as insect antifeedant and antifungal [7]. Additional endemic plants with reported insect antifeedant effects included species of the genus *Bethencourtia* [12,13]. This genus, endemic to the Canary Islands, consists of three species: *Bethencourtia hermosae* (Pit), *Bethencourtia palmensis* (Nees) Choisy, and *Bethencourtia rupicola* (B. Nord.) B. Nord [14]. Previous studies on *B. palmensis* showed the presence of silphinene sesquiterpenes with strong insect antifeedant effects acting on insect γ-aminobutyric acid (GABA) receptors [13]. Therefore, the plant *B. palmensis* was chosen for the isolation of fungal endophytes with the ability to produce secondary metabolites with biopesticidal properties (as antifungal, nematicidal, insect antifeedant, and ixodicidal agents).

In this work, we report on the isolation of the endophytic fungal strain SPH2 from *Bethencourtia palmensis* and the isolation and characterization of the bioactive compounds (against the fungal pathogens *Alternaria alternata*, *Fusarium oxysporum,* and *Botrytis cinerea* and the tick *Hyalomma lusitanicum*). Additionally, a metabolomic study of the SPH2 time-course fermentation has been carried out.

## 2. Materials and Methods

### 2.1. Plant Material

*Bethencourtia palmensis* was collected in Barranco del Rio, Abona (Tenerife, Spain) (28°34′10″ N 16°18′48″ W). The samples were placed into sterile polybags and transported, under refrigeration, in a box container until isolation processing within 48 h of collection.

### 2.2. Isolation of Endophytic Fungus SPH2

Endophytic fungi were isolated according to Kumar et al. (2013) [15]. The stem surface was sterilized with 70% ethanol for 2 min followed by 1% sodium hypochlorite for 3 min. Sterilized stems were dried on a sterile blotting sheet and then chopped in a sterile Petri plate and transferred to Potato Dextrose Agar plates (PDA) with 50 mg/L of chloramphenicol to inhibit bacterial growth. These plates were incubated at 24 °C for 3–15 days in a growth chamber. The fungal colonies were transferred to fresh PDA plates to get pure cultures. SPH2 was initially identified by microscopic examination and later identified by molecular methods. The culture was maintained on PDA by routine subculturing.

### 2.3. Molecular Characterization of SPH2

The genomic DNA of the pure fungal isolate SPH2 was extracted using DNeasy Plant mini kit (Qiagen GmbH, Hilden, Germany, Cat. No 69104) by following the manufacturer’s instructions. The extracted DNA was used for PCR amplification by primer ITS1 (5′-TCCGTAGGTGAACCTGCGG-3′) and ITS4 (5′-TCCTCCGCTTATTGATATGC-3′) according to Kumar et al. (2011) [16]. Genomic DNA (100–200 ng) was amplified on a PTC-200 Thermal Cycler (MJ Research, San Diego, CA, USA) in a 25 µL final volume with the AmpONE Taq DNA polymerase PCR kit (GeneAll, Seoul, Korea) for 35 cycles (95 °C, 1 min; 50 °C, 20 s; 72 °C, 1.5 min) after an initial denaturation (95 °C, 2 min) and followed by a final extension (72 °C, 7 min). Amplicons were checked by agarose gel (1%) electrophoresis, purified using the EXO-SAP-IT kit (Affimetrix-USB; Thermo Fisher Scientific, Waltham, MS, USA), and sequenced on an AB 3500 Genetic Analyzer (Thermo Fisher Scientific, Waltham, MS, USA) at the University of La Laguna (La Laguna, Spain) genomic service. A BLASTN search of the sequence against the NCBI nucleotide identified strain SPH2 as *Aspergillus sp.,* similar to these in the group Circumdati (*A. ochraceus,* GENBANK accession number KX901282.1 and *A. westerdijkiae,* GENBANK accession number KY608057.1).

### 2.4. Cultivation of SPH2 for Extract Preparation

SPH2 was cultivated on PDA solid medium for 8 days at 25 °C. Sterile water (10 mL) was added to each Petri dish to obtain a suspension of superficial mycelium and then transferred to an Erlenmeyer flask (250 mL) with 50 mL of a modified Czapek-Dox-Yeast liquid media ([Cz-L: NaNO3 (2 g/L), KH2PO4 (5 g/L), MgSO4 (0.5 g/L), FeSO4 (0.01 g/L), ZnSO4 (0.003 g/L), yeast extract (1 g/L) and glucose (60 g/L)]. This suspension was cultivated for 3 days at 25 °C under continuous agitation and was used as pre-inoculum. Two Erlenmeyer flasks (500 mL) with 200 mL of fresh Cz-L medium each were inoculated with 15 mL of pre-inoculum and incubated at 25 °C under continuous agitation for 4 and 10 days.

### 2.5. Extract Preparation

The culture media was filtered through a 25 µm pore diameter cheesecloth using a Buchner funnel to separate the mycelium, submitted to exhaustive liquid/liquid extraction with ethyl acetate (EtOAc), dried over SO_4_Na_2_ and concentrated under reduced pressure to give crude SPH2 extracts (yield of 177 or 660 mg/L for 4 and 10 days, respectively) for bioguided fractionation.

### 2.6. Time-Course Fermentation

A total of 27 Erlenmeyer flasks were prepared with 100 mL of culture media (three replicates/sample) and inoculated with 500 mg mycelium from the pre-inoculum suspension. Three flasks were sampled on days 1, 3, 4, 6, 7, 8, 10, 12, and 13 of incubation. The culture media was separated from the mycelium by filtration with a Buchner filter and the mycelium from each sample was frozen with liquid nitrogen, lyophilized, and weighed.

### 2.7. Bioassays

#### 2.7.1. Antifungal Bioassay

The fungal species *Fusarium moniliforme, F. solani, F. oxysporum, Alternaria alternata,* and *Botrytis cinerea* came from the fungal collection at Instituto de Productos Naturales y Agrobiologia-CSIC (Santa Cruz de Tenerife, Spain). The mycelial growth inhibition test was carried out in 12-well plates (Falcon) by a modified agar-dilution method with the addition of 0.05 mg/mL of methyltetrazolium salts (MTT). Extracts and pure compounds dissolved in ethanol (EtOH) were tested at different concentrations (extracts at 1, 0.5, 0.25 and 0.1 mg/mL; compounds at 0.5, 0.1, 0.25, and 0.05 mg/mL) and were incorporated into the culture medium before plates were poured. A series of test solutions into PDA (potato dextrose agar) and MTT were prepared for each concentration tested and then 300 µL were added to each well. All treatments were replicated four times and EtOH was used as a negative control. After incubation in darkness at 27 °C for 48 h, fungal colonies were digitalized and measured with the application ImageJ (http://imagej.nih.gov/ij/). Percent inhibition (%I) was calculated as: %I = (C − T/C) × 100, where C is the diameter of the control colonies and T is the diameter of the test colonies [17]. Data were analyzed with Statgraphics statistical analysis software (Centurion XVIII) and EC_50_ values (effective dose to obtain 50% of inhibition) were determined by means of a regression curve mycelial growth inhibition versus log dose.

#### 2.7.2. Ixodicidal Activity

*Hyalomma lusitanicum* engorged female ticks were collected in central Spain (Finca La Garganta, Ciudad Real) from their host (deer) and maintained at 22–24 °C and 70% relative humidity until oviposition and egg hatch. The resulting larvae (4–6 weeks old) were used for the bioassays [18]. Briefly, 50 µL of test solution was added to 25 mg of powdered cellulose at different concentrations and the solvent was evaporated. For each test, three replicates with 20 larvae each were used. Dead ticks were counted using a binocular magnifying glass 24 h after contact with the treated cellulose under the environmental conditions described. Larvicidal activity data are presented as percent mortality corrected according to Schneider-Orelli’s formula. Effective lethal doses (LC_50_ and LC_90_) were calculated by Probit Analysis (5 serial dilutions, STATGRAPHICS Centurion XVI, version 16.1.02).2.10. 

#### 2.7.3. Insecticidal and Nematicidal Activities

See Supplementary Materials for material and methods.

### 2.8. Isolation and Identification of the Bioactive Compounds

Column chromatography (CC): Sephadex LH-20 (Sigma–Aldrich). TLC: silica gel (105554 and 105744; Merck); compounds were visualized on TLC under a UV lamp and óleum solution. Preparative HPLC: Varian Prostar 210 HPLC Pump equipped with normal phase (Ace 5 SIL 127-2510) and reverse phase (Interchrom KR/25M Kromasil 4g Si) columns. Gas chromatography–mass spectrometry (GC-MS): Shimadzu GC-2010 gas chromatograph coupled to a Shimadzu GCMS-QP2010 Ultra mass detector (electron ionization, 70 eV) and equipped with a 30 m × 0.25 mm i.d. capillary column (0.25 µm film thickness) Teknokroma TRB-5 (95%) Dimethyl-(5%) dimethylpolysiloxane. NMR Spectra: Bruker AMX 500 MHz spectrometer with pulsed-field gradient, solvent as internal standard (CDCl_3_, at δ_H_ 7.26 and δ_C_ 77.0); the programs used for DEPT, ^1^H, ^1^H-COSY, NOESY, HSQC, and HMBC experiments included in the Bruker software. EI and HR-ESI-MS: Micromass Autospec and Micromass LCT Premier spectrometers in m/z.

### 2.9. Bioguided Extract Fractionation

An antifungal EtOAc extract (4 days, 146 mg) was chromatographed on a Sephadex LH-20 column (140 mL) eluted with n-hexane:Cl_2_CH_2_:MeOH (2:1:1) mixture to afford two fractions. The active fraction 1 (20 mg) was further purified by semi-preparative HPLC (Ultrasphere ODS, 10.0 × 250 mm, 5 μm) eluted with an isocratic mixture of MeOH:H_2_O (80:20) at a flow rate of 3 mL/min to yield compound **2** (6 mg).

### 2.10. Compound Identification

Compound **1** was analyzed by GC-MS with the following working conditions: split ratio (20:1), injector temperature 300 °C, temperature of the transfer line connected to the mass spectrometer 250 °C, initial column temperature 70 °C, then heated to 290 °C at 6°/min. Electron ionization mass spectra and retention data were used to assess the identity of compounds by comparing them with those of standards or found in the Wiley 229 and NIST Mass Spectral Database. Further, the retention times of authentic compound (isolated in this work and purchased from Cayman Chemical Company (Ann Arbor, MI, USA) were also used to confirm the identity of the compound. Compound **1** was identified as mellein.

*(−)-Mellein* (**1**)**:** [α]_D_ -77.5 (c 0.08, CHCl_3_); ^1^H NMR (500 MHz, CDCl_3_), δ: 11.02 (1H, s, -OH), 7.41 (1H, t, *J* = 7.9 Hz, H-6), 6.89 (1H, d, *J* = 8.4 Hz, H-7), 6.69 (1H, d, *J* = 7.4 Hz, H-5), 4.74 (1H, dq, J = 12.7, 6.4 Hz, H-3), 2.93 (2H, dd, *J* = 7.3, 2.1 Hz, H-4), 1.54 (3H, d, *J* = 6.3 Hz,-CH_3_). ^13^C NMR (125 MHz, CDCl_3_), δ: 169.9 (C-1), 162.2 (C-8), 139.4 (C-10), 136.1 (C-6), 117.9 (C-5), 116.2 (C-7), 108.3 (C-9), 76.1 C-3), 34.6 (C-4), 20.7 (-CH_3_).

Compounds **2** and **3** were identified as neoaspergillic acid and neohydroxyaspergillic acid. Their molecular formulas (C_12_H_20_N_2_O_2_ and C_12_H_20_N_2_O_3_) were determined by ESI-HREIMS (m/z 223.1447 [M-H]^+^ and 263.367[M + Na]^+^). Their ^1^H NMR and ^13^C NMR spectra are shown in Table 1).

### 2.11. Compound Quantification

GC-MS analysis of the extracts from the time-course fungal fermentation was carried out at the same working conditions as above. Electron ionization mass spectra and retention data were used to assess the identity of compounds by comparing them with those of standards or found in the Wiley 229 and NIST Mass Spectral Database. Further, the retention times of authentic compounds (isolated in this work) were also used to confirm the identity compounds **1**–**3**. Extract solutions (4 µg/µL) were dissolved in dichloromethane (DCM) for sample injection (1 µL/injection). The concentrations of **1**–**3** were calculated based on a calibration curve built with serial dilutions (1.0, 0.5, 0.25, 0.125, and 0.0625 µg/µL) of an external standard (bornyl acetate, Sigma Aldrich) and their peak area without a correction factor.

The presence of the mycotoxin ochratoxin A was verified by analyzing the time-course extracts by HPLC-MS on a Shimadzu LC-20AD HPLC coupled to a LCMS-2020 QP mass spectrometer using an electrospray ionization (ESI) interface and CTO-10AS VP column oven. Sample injections (5 μL) were carried out by a SIL-20A XR autosampler. Samples were separated in an ACE 3 C18 column (150 × 4.6 mm, 3 µm particle size) and ACE 3 C18 analytical guard cartridge at 25 °C. The mobile phase consisted of (A) MiliQ water and containing 0.1% acetic acid and (B) methanol (HPLC-MS grade) and containing 0.1% acetic acid. The solvent gradient was 38–100% B in 45 min, 100% B for 10 min, and 38% B for 7 min. Solvent and N (drying gas) flow rates were 0.5 mL/min and 15 L/min, respectively. The electrospray capillary potential was set to +4.50 kV and ESI was conducted in the Full Scan positive mode (m/z = 100–700) with a potential of 1.40 kV and a capillary temperature of 250 °C. Extract (0.25 μg/μL) stock solutions were dissolved in MeOH for sample injection. Commercial ochratoxin A (Sigma Aldrich) was injected at a concentration of 0.05 μg/μL.

## 3. Results

In this work the endophytic fungus SPH2 was isolated from a stem portion of the endemic plant *Bethencourtia palmensis* and identified as *Aspergillus* sp., isolate SPH2, similar to these in the group Circumdati (*A. ochraceus y A. westerdijkiae*).

### 3.1. Fermentation and Compound Identification

**Figure 1:**
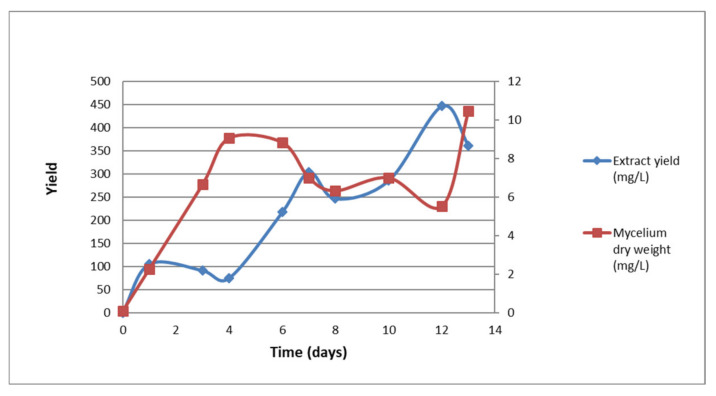
Time course of SPH2 mycelial and extract yield Table 1.

The time-course fermentation of SPH2 showed a maximum of mycelial weight at days 4 and 13, reaching the stationary phase by day 7. The extract yield reached a stable production by day 7 with a second increase on day 12 (Figure 1).

The ethyl acetate extracts from SPH2 were screened for their biocontrol properties against plant pathogens (*Fusarium moniliforme*, *Alternaria alternata,* and *Botrytis cinerea*) (Table 2 and Table 3), insect pests (*Spodoptera littoralis*, *Myzus persicae*, *Rhopalosiphum padi*) (Table S1) a plant parasitic nematode (*Meloidogyne javanica*) (Table S2) and ticks (*Hyalomma lusitanicum*) (Table 2). Extracts from days 3–13 showed significant fungicidal and ixodicidal effects. The antifungal effects on *B. cinerea* and *A. alternata*, the most sensitive species, were stronger between days 3 and 7 of incubation, while the ixodicidal effects started on day 7 (the most active extract) and were maintained until day 13 (Table 2). These extracts were not antifeedant or nematicidal (Tables S1 and S2).

**Figure 2:**
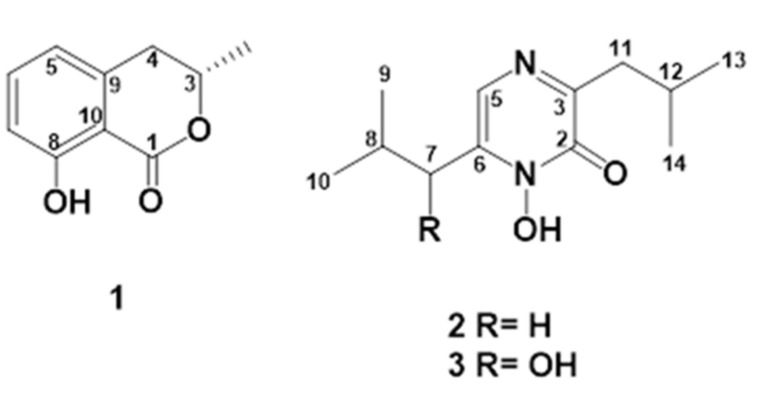
Chemical structures of compounds **1**–**3**.

The bioguided fractionation of SPH2 extracts resulted in the isolation of compounds **1**–**3** (Figure 2). Compound **1** was identified as mellein based on its GC-MS and spectroscopic data [19,20]. Compounds **2** and **3**, previously isolated as natural products from *Aspergillus ochraceus* [21], were identified as neoaspergillic and neohydroxyaspergillic acids, respectively, based on their spectroscopic data [22,23,24].

Compound **2** (neoaspergillic acid) was an effective antifungal against *A. alternata, B. cinerea,* and *F. oxysporum,* respectively, followed by mellein (**1**) (Table 3).

Mellein (**1**), isolated from the ixodicidal fraction, showed a strong effect against *H. lusitanicum* larvae (LD_50_ = 0.48 µg/mg, 0.44–0.51 95% Confidence Limits), being 10 times more effective than thymol (LD_50_ = 2.94 µg/mg, 2.08–3.54 95% Confidence Limits).

### 3.2. Quantification of 1–3 and Metabolomics

**Figure 3:**
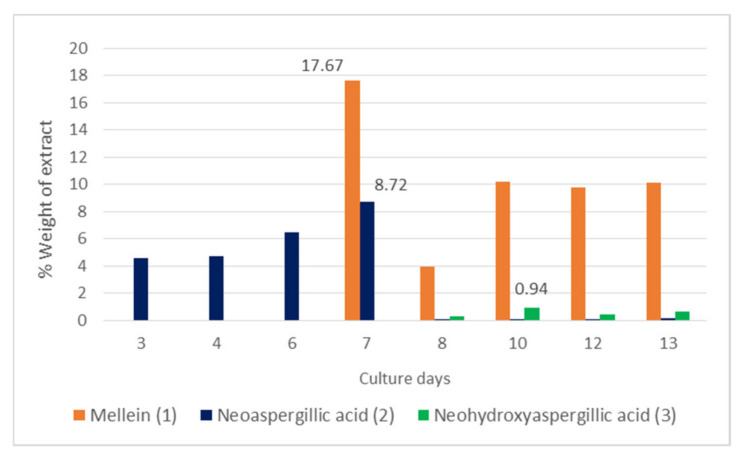
Time-course production of compounds **1**–**3**.

The time-course production of **1**–**3** was quantified by GC-MS. Neoaspergillic acid (**2**) was the first compound detected (days 3 to 7), ranging between 4.11–11.7% weight, with the highest content found for day 7 (Figure 3). Neohydroxyaspergillic acid (**3**) appeared between days 8–13, ranging between 0.3–0.9% weight, with the highest content found for day 10. Mellein (**1**) was present between days 7–13 ranging between 3.9–17.7% weight, with the highest content found for day 7 (Figure 3). Additional analysis of these extracts carried out by HPLC-MS showed no presence of ochratoxin A.

## 4. Discussion

In this work, the endophytic fungus SPH2 has been isolated from the plant *Bethencourtia palmensis* and identified as *Aspergillus sp.* based on the similarity of the SPH2 ITS with isolates from the group Circumdati of *Aspergillus* (*A. ochraceus, A. westerdijkiae*).

The genus *Aspergillus* is one of the most common among endophytic fungi associated with marine and terrestrial hosts. Endophytic *Aspergillus* have been described from the arctic tundra to the tropics [1]. Specifically, *A. ochraceus* has been isolated from the fern *Selaginella stauntoniana* [25]; a wide range of plants including *Euphorbia geniculata* [26] *Polygonatum cyrtonema* [27], alfalfa (*Medicago sativa*) [28], bamboo [29], the cactus *Cereus jamacaru* [30], *Bauhinia forficata* [31]; orchids (*Bulbophyllum neilgherrense and Vanda testacea*) [32]; and *Catha edulis* [33]. *A. ochraceus* has also been isolated as an endosymbiont from marine organisms such as the coral *Dichotella gemmacea* [34] and the marine brown alga *Sargassum kjellmanianum* [35,36].

The time-course fermentation of SPH2 gave extracts with strong fungicidal and ixodicidal effects at different fermentation times. The bioguided isolation of these extracts gave compounds **1**–**3**. Mellein (**1**) belongs to the subgroup of 3,4-dihydroisocoumarins. *R*-(-)-mellein is produced by fungi, plants insects, and bacteria, with fungi being the main natural source of this compound [37]. Overall, eleven out of 27 species in the *Aspergillus* section Circumdati produce mellein [38]. Among the different biological activities of **1**, phytotoxicity and antifungal are the main ones reported [37]. In this work, mellein (**1**) showed moderate antifungal effects against *F. oxysporum, A. alternata,* and *B. cinerea*. Similarly, previous reports showed moderate activity of **1** against *B. cinerea* [19,39,40], *F. oxysporum,* and *F. solani* [41]. Mellein (**1**) also showed potent ixodicidal effects against larvae of the hard tick *Hyalomma lusitanicum*. In arthropods, **1** seems to play an ecological role for some insect species: as an attractant to the beetle *Tribolium confusum* [42], and as a defensive exudate [43,44]. Furthermore, it has been suggested that the termite *Reticulitermes speratus* used **1** as an antifungal protectant [45]. On the other hand, **1** has been described as being larvicidal against *Aedes aegypti* [46]. However, this is the first report on the ixodicidal effects of mellein (**1**). Ticks are the second most important group of disease vectors after mosquitoes because they can transmit a great variety of pathogens to humans and animals and are in expansion due to climate change [47]. For example, *Hyalomma* ticks, vectors of the Crimea Congo Hemorrhagic Fever virus, have spread from their original distribution (African and Mediterranean environments) to other European countries, becoming an increasing public health concern [48,49,50,51]. Therefore, new effective and safer tick control agents are needed.

Compound **2**, with antifungal effects, was identified as neoaspergillic acid and compound **3** as hydroxyneoaspergillic acid. Neoaspergillic and hydroxyneoaspergillic acids (**2,3**) have been isolated from members of *Aspergillus* section Circumdati and related to the presence of homologs of the aspergillic acid gene cluster 11, responsible for their biosynthesis [52]. The aspergillic acid group of mycotoxins includes a number of closely related pyrazine metabolites with antibiotic properties [53]. Neoaspergillic acid (**2**) has been reported to have antitumoral [54,55] and antibacterial effects [56,57]. Compound **2** has also been reported to have weak antifungal effects (against *Candida albicans* and *Aspergillus terreus*) [54]. This work has shown potent antifungal effects of compound **2** against phytopathogenic fungi (*Alternaria alterna, Botrytis cinerea,* and *Fusarium oxysporum*) for the first time.

The time-course quantification of **1**–**3** showed that the pyrazine neoaspergillic acid (**2**) was the major compound that appeared during the exponential phase of the fungal growth (days 3–7), while neohydroxyaspergillic acid (**3**) appeared during the stationary phase (days 8–13), suggesting that **2** is the biosynthetic precursor of **3.** We conclude that exponential phase extracts were antifungal because of their content in **2**. Mellein (**1**) was detected during days 7–13. These extracts were antifungal and effective against the tick *Hyalomma lusitanicum* because of their content in mellein (**1**). *Aspergillus* species section Circumdati, produce ochratoxin A, a nephrotoxic mycotoxin [38,57]. However, in this study, we have not detected ochratoxin A by HPLC-MS in SPH2 extracts.

## 5. Conclusions

In this work, the endophytic fungus SPH2 has been isolated from the plant *Bethencourtia palmensis* and identified as *Aspergillus sp.* The time-course fermentation of SPH2 gave extracts with strong fungicidal and ixodicidal effects at different fermentation times. The bioguided isolation of these extracts gave compounds **1**–**3**. Mellein (**1**) was shown for the first time to have strong ixodicidal effects, in addition to being fungicidal. Neoaspergillic acid (**2**) showed potent antifungal effects against *Alternaria alterna, Botrytis cinerea,* and *Fusarium oxysporum*. Compound **2** appeared during the exponential phase of fungal growth while neohydroxyaspergillic acid **3** appeared during the stationary phase, suggesting that **2** is the biosynthetic precursor of **3**.

The toxin ochratoxin A was not detected. Therefore, the fungal endophyte SPH2 could be a biotechnological tool for the production of ixodicidal mellein-rich extracts.

## Figures and Tables

**Table 1:** 1H NMR and 13C NMR spectra of compounds **2** and **3** in CD3OD.

Position	2		3	
	*δ*_H_ (*J* in Hz)	*δ*_c_, Mult	*δ*_H_ (*J* in Hz)	*δ*_c_, Mult
2		158.8, C		158.2, C
3		148.5, C		149.2, C
5	7.26, s	126.9, CH	7.49, s	125.7, CH
6		142.5, C		143.4, C
7	2.63, d (7.1)	38.1, CH_2_	4.75, br s	74.0, CH
8	2.19,dq (13.8, 6.9)	26.4, CH	2.32, br s	32.5, CH
9	0.89, d (6.6)	22.9, CH_3_	0.90, m	19.2, CH_3_
10	0.89, d (6.6)	22.9, CH_3_	0.90, m	17.7, CH_3_
11	2.60, d (7.3)	42.6, CH_2_	2.64, d (7.3)	42.6, CH_2_
12	2.11, dt (13.6, 6.8)	28.4, CH	2.14, br s	28.6, CH
13	0.89, d (6.6)	22.9, CH_3_	0.90, m	22.9, CH_3_
14	0.89, d (6.6)	22.9, CH3	0.90, m	22.9, CH3

**Table 2:** Ixodicidal activity on *Hyalomma lusitanicum* larve (mortality) and antifungal (mycelial growth inhibition) on *Fusarium oxysporum, Alternaria alternata, Botrytis cinerea*, of the time-course SPH2 extracts.

Days	*H. Lusitanicum*	*F. Oxysporum*	*A. Alternata*	*B. Cinerea*
	LD_50_ (µg/mg) ^a^	EC_50_ (mg/mL) ^b^		
3	>20	0.27 (0.24–0.30)	0.10 (0.07–0.13)	0.04 (0.01–0.12)
4	>20	0.25 (0.20–0.31)	0.14 (0.11–0.18)	0.05 (0.02–0.10)
6	>20	0.31 (0.26–0.38)	0.16 (0.12–0.21)	0.03 (0.02–0.05)
7	2.85 (2.61, 3.18)	0.45 (0.31–0.65)	0.06 (0.05–0.09)	0.02 (0.01–0.05)
8	7.82 (6.91, 8.75)	0.31 (0.25–0.39)	0.25 (0.18–0.34)	0.12 (0.08–0.18)
10	6.63 (5.92–7.49)	0.21 (0.18–0.25)	0.16 (0.12–0.22)	0.16 (0.12–0.22)
12	3.30 (3.09, 3.52)	0.39 (0.31–0.48)	0.24 (0.20–0.29)	0.24 (0.20–0.29)
13	7.18 (6.67, 7.78)	0.87 (0.62–1.24)	0.44 (0.37–0.51)	0.22 (0.19–0.26)

^a^ Lethal dose calculated by Probit analysis; ^b^ Effective doses calculated by linear regression.

**Table 3:** Antifungal effects (mycelial growth inhibition) of compounds **1**–**3**.

Compound	Effective Doses (mg/mL) ^a^	Mycelial Growth Inhibition		
		*F. Oxysporum*	*A. Alternata*	*B. Cinerea*
**1**	EC_50_	0.34 (0.27–0.43)	0.44 (0.34–0.57)	0.29 (0.23–0.37)
**2**	EC_50_	0.07 (0.04–0.10)	0.01 (0.00–0.02)	0.04 (0.03–0.04)
**3**	EC_50_	>0.5	0.20 (0.17–0.25)	>0.5

^a^ Effective doses calculated by linear regression.

## Data Availability

The experimental data is available at the Biopesticides Group-CSIC databases (contact: azu@ica.csic.es).

## Supplementary Materials

The following are available online at https://www.mdpi.com/2309-608X/7/2/109/s1: Material and Methods S1, Table S1(Antifeedant effects of SPH2 extract), Table S2 (Nematicidal activity of SPH2 extract).

## References

[1] Tawfike A.F., Tate R., Abbott G., Young L., Viegelmann C., Schumacher M., Han B.W., Edrada-Ebel R. (2017). Metabolomic Tools to Assess the Chemistry and Bioactivity of EndophyticAspergillusStrain. Chem. Biodivers..

[2] Strobel G., Daisy B. (2003). Bioprospecting for Microbial Endophytes and Their Natural Products. Microbiol. Mol. Biol. Rev..

[3] Firáková S., Šturdíková M., Múčková M. (2007). Bioactive secondary metabolites produced by microorganisms associated with plants. Biologia.

[4] Germaine K., Keogh E., Garcia-Cabellos G., Borremans B., Van Der Lelie D., Barac T., Oeyen L., Vangronsveld J., Moore F.P., Moore E.R. (2004). Colonisation of poplar trees by gfp expressing bacterial endophytes. FEMS Microbiol. Ecol..

[5] Wang Y., Dai C.-C. (2011). Endophytes: A potential resource for biosynthesis, biotransformation, and biodegradation. Ann. Microbiol..

[6] Guo B., Wang Y., Sun X., Tang K. (2008). Bioactive natural products from endophytes: A review. Appl. Biochem. Microbiol..

[7] Andrés M.F., Diaz C.E., Giménez C., Cabrera R., González-Coloma A. (2017). Endophytic fungi as novel sources of biopesticides: The Macaronesian Laurel forest, a case study. Phytochem. Rev..

[8] Morales-Sánchez V., Fe Andrés M., Díaz C.E., González-Coloma A. (2020). Factors Affecting the Metabolite Productions in Endophytes: Biotechnological Approaches for Production of Metabolites. Curr. Med. Chem..

[9] Higginbotham S., Arnold A.E., Ibañez A., Spadafora C., Coley P.D., Kursar T.A. (2013). Bioactivity of Fungal Endophytes as a Function of Endophyte Taxonomy and the Taxonomy and Distribution of Their Host Plants. PLoS ONE.

[10] Harrison J.G., Griffin E.A. (2020). The diversity and distribution of endophytes across biomes, plant phylogeny and host tissues: How far have we come and where do we go from here?. Environ. Microbiol..

[11] The Macaronesian Region. https://ec.europa.eu/environment/nature/natura2000/biogeog_regions/macaronesian/index_en.htm.

[12] Fraga B.M., Díaz C.E., Amador L.J., Reina M., Santana O., Gonzalez-Coloma A. (2014). Bioactive compounds from transformed root cultures and aerial parts of *Bethencourtia hermosae*. Phytochemistry.

[13] Portero A.G., González-Coloma A., Reina M., Díaz C.E. (2012). Plant-defensive sesquiterpenoids from *Senecio* species with biopesticide potential. Phytochem. Rev..

[14] Nordenstam B. (2006). “Canariothamnus B.” Nord., a new genus of the Compositae-Senecioneae, endemic to the Canary Islands. Comp. Newsl..

[15] Kumar S., Kaushik N. (2013). Endophytic Fungi Isolated from Oil-Seed Crop Jatropha curcas Produces Oil and Exhibit Antifungal Activity. PLoS ONE.

[16] Kumar S., Kaushik N., Edrada-Ebel R., Ebel R., Proksch P. (2011). Isolation, characterization, and bioactivity of endophytic fungi of Tylophora indica. World J. Microbiol. Biotechnol..

[17] Parra A.J.E., Cuca L.E., González-Coloma A. (2019). Antifungal and phytotoxic activity of benzoic acid derivatives from inflorescences of *Piper cumanense*. Nat. Prod. Res..

[18] Ruiz-Vásquez L., Olmeda A.S., Zúñiga G., Villarroel L., Echeverri L.F., González-Coloma A., Reina M. (2017). Insect Antifeedant and Ixodicidal Compounds from Senecio adenotrichius. Chem. Biodivers..

[19] Wang J., Wang G., Zhang Y., Zheng B., Zhang C., Wang L. (2014). Isolation and identification of an endophytic fungus *Pezicula* sp. in *Forsythia viridissima* and its secondary metabolites. World J. Microbiol. Biotechnol..

[20] Cimmino A., Cinelli T., Masi M., Reveglia P., Da Silva M.A., Mugnai L., Michereff S.J., Surico G., Evidente A. (2017). Phytotoxic Lipophilic Metabolites Produced by Grapevine Strains of *Lasiodiplodia* Species in Brazil. J. Agric. Food Chem..

[21] Yamazaki M., Maebayashi Y., Miyaki K. (1972). Isolation of a new type of pyrazine metabolite from *Aspergillus* sp.. Chem. Pharm. Bull..

[22] Maebayashi Y., Sumita M., Fukushima K., Yamazaki M. (1978). Isolation and structure of red pigment from *Aspergillus* sp. Wilh. Chem. Pharm. Bull..

[23] Assante G., Camarda L., Locci R., Merlini L., Nasini G., Papadopoulos E. (1981). Isolation and structure of red pigments from Aspergillus flavus and related species, grown on a differential medium. J. Agric. Food Chem..

[24] Bao J., Wang J., Zhang X.Y., Nong X.H., Qi S.H. (2017). New furanone derivatives and alkaloids from the co-culture of marine derived fungi *Aspergillus sclerotiorum* and *Penicillium citrinum*. Chem. Biodivers..

[25] Luo P., Shao G., Zhang S., Zhu L., Ding Z., Cai L. (2020). Secondary metabolites of endophytic fungus *Aspergillus sp.* SX-C7 from *Selaginella stauntoniana*. Zhong Cao Yao.

[26] Kamel N.M., Abdel-Motaal F.F., El-Zayat S.A. (2020). Endophytic fungi from the medicinal herb *Euphorbia geniculata* as a potential source for bioactive metabolites. Arch. Microbiol..

[27] Cheng Z., Ke Z., Wu Y. (2019). Study on secondary metabolites of endophytic fungus *Aspergillus sp.* from *Polygonatum cyrtonema*. Zhong Cao Yao.

[28] Attia E.Z., Farouk H.M., Abdelmohsen U.R., El-Katatny M.H. (2020). Antimicrobial and extracellular oxidative enzyme activities of endophytic fungi isolated from alfalfa (*Medicago sativa*) assisted by metabolic profiling. S. Afr. J. Bot..

[29] Paynor K.A., David E.S., Valentino M.J.G. (2016). Endophytic fungi associated with bamboo as possible sources of single cell protein using corn cob as a substrate. Mycosphere.

[30] Da Silva D.M., de Sousa Carvalho F.R., Moura A.G., Martins L., Ferreira P.M., Peron A.P. (2015). Cytotoxic action of the stem aqueous extract of the stem of *Cereus jamacaru* DC. (mandacaru). Rev. Cuba. Plantas Med..

[31] Bezerra J.D., Nascimento C.C., Barbosa R.D.N., Da Silva D.C., Svedese V.M., Silva-Nogueira E.B., Gomes B.S., Paiva L.M., Motta C.M.D.S. (2015). Endophytic fungi from medicinal plant Bauhinia forficata: Diversity and biotechnological potential. Braz. J. Microbiol..

[32] Sudheep N.M., Sridhar K.R. (2012). Non-mycorrhizal fungal endophytes in two orchids of Kaiga forest (Western Ghats), India. J. For. Res..

[33] Mahmoud A.-L.E. (2000). Mycotoxin-producing potential of fungi associated with qat (Catha edulis) leaves in yemen. Folia Microbiol..

[34] Guo S., Mao W., Yan M., Zhao C., Li N., Jimiao S., Lin C., Liu X., Guo T., Guo T. (2014). Galactomannan with novel structure produced by the coral endophytic fungus *Aspergillus* sp.. Carbohydr. Polym..

[35] Cui C.M., Li X.M., Meng L., Li C.S., Huang C.G., Wang B.G. (2010). 7-Nor-ergosterolide, a pentalactone-containing norsteroid and related steroids from the marine-derived endophytic *Aspergillus* sp. EN-31. J. Nat. Prod..

[36] Cui C.M., Li X.M., Li C.S., Sun H.F., Gao S.S., Wang B.G. (2009). Benzodiazepine Alkaloids from Marine-Derived Endophytic Fungus Aspergillus sp.. Helv. Chim. Acta.

[37] Reveglia P., Masi M., Evidente A. (2020). Melleins—Intriguing Natural Compounds. Biomolecules.

[38] Visagie C., Varga J., Houbraken J., Meijer M., Kocsubé S., Yilmaz N., Fotedar R., Seifert K., Frisvad J., Samson R. (2014). Ochratoxin production and taxonomy of the yellow aspergilli (*Aspergillus* section Circumdati). Stud. Mycol..

[39] Zhao J.H., Zhang C., Wang L.W., Wang J.Y. (2012). Bioactive secondary metabolites from Nigrospora sp. LLGLM003, an endophytic fungus of the medicinal plant Moringa oleifera Lam. World J. Microbiol. Biotechnol..

[40] Cimmino A., Maddau L., Masi M., Linaldeddu B.T., Evidente A. (2019). Secondary metabolites produced by Sardiniella urbana, a new emerging pathogen on European hackberry. Nat. Prod. Res..

[41] Wang Q., Yang X.-Q., Miao C.-P., Xu L.-H., Ding Z., Yang Y.-B., Zhao L.-X. (2018). A New Pair of Pentaketide Diastereoisomers from Aspergillus melleus YIM PHI001. Rec. Nat. Prod..

[42] Hori M., Aoki Y., Shinoda K., Chiba M., Sasaki R. (2019). Wood volatiles as attractants of the confused flour beetle, *Tribolium confusum* (Coleoptera: Tenebrionidae). Sci. Rep..

[43] Voegtle H.L., Jones T.H., Davidson D.W., Snelling R.R. (2008). E-2-Ethylhexenal, E-2-Ethyl-2-Hexenol, Mellein, and 4-Hydroxymellein in Camponotus Species from Brunei. J. Chem. Ecol..

[44] Blum M.S., Morel L., Fales H.M. (1987). Chemistry of the mandibular gland secretion of the ant Camponotus vagus. Comp. Biochem. Physiol. Part B Comp. Biochem..

[45] Mitaka Y., Mori N., Matsuura K. (2019). A termite fungistatic compound, mellein, inhibits entomopathogenic fungi but not egg-mimicking termite ball fungi. Appl. Èntomol. Zool..

[46] Kendagor A.C., Langat M.K., Cheplogoi P.K., Omolo J.O. (2013). Larvicidal activity of mellein from cultures of an ascomycete *Pezicula livida* against *Aedes aegypti*. Int. J. Life Sci. Biotechnol. Pharma. Res..

[47] Sajid M., Kausar A., Iqbal A., Abbas H., Iqbal Z., Jones M. (2018). An insight into the ecobiology, vector significance and control of *Hyalomma* ticks (Acari: Ixodidae): A review. Acta Trop..

[48] Chitimia-Dobler L., Schaper S., Rieß R., Bitterwolf K., Frangoulidis D., Bestehorn M., Springer A., Oehme R.M., Drehmann M., Lindau A. (2019). Imported Hyalomma ticks in Germany in 2018. Parasites Vectors.

[49] Hansford K.M., Carter D., Gillingham E.L., Hernandez-Triana L.M., Chamberlain J., Cull B., McGinley L., Phipps L.P., Medlock J.M. (2019). Hyalomma rufipes on an untraveled horse: Is this the first evidence of Hyalomma nymphs successfully moulting in the United Kingdom?. Ticks Tick-Borne Dis..

[50] Buczek A.M., Buczek W., Bartosik K. (2020). The Potential Role of Migratory Birds in the Rapid Spread of Ticks and Tick-Borne Pathogens in the Changing Climatic and Environmental Conditions in Europe. Int. J. Environ. Res. Public Health.

[51] Grandi G., Chitimia-Dobler L., Choklikitumnuey P., Strube C., Springer A., Albihn A., Jaenson T., Omazic A. (2020). First records of adult *Hyalomma marginatum* and *H. rufipes* ticks (Acari: Ixodidae) in Sweden. Ticks Tick-Borne Dis..

[52] Lebar M.D., Mack B.M., Carter-Wientjes C.H., Gilbert M.K. (2019). The aspergillic acid biosynthetic gene cluster predicts neoaspergillic acid production in *Aspergillus* section Circumdati. World Mycotoxin J..

[53] Xu X.-Y., He F., Zhang X., Bao J., Qi S. (2013). New mycotoxins from marine-derived fungus *Aspergillus* sp. SCSGAF0093. Food Chem. Toxicol..

[54] Chen X.W., Li C.W., Hua W., Wu C.J., Cui C.B., Zhu T.J., Gu Q.Q. (2013). Metabolites of *Aspergillus* sp. 16-02-1 isolated from a deep sea sediment and preliminary test of their antitumor and antifungal activities. Chin. J. Mar. Drugs.

[55] Cardoso-Martínez F., de la Rosa J.M., Díaz-Marrero A.R., Darias J., D’Croz L., Cerella C., Diederich M., Cueto M. (2015). Oximoaspergillimide, a fungal derivative from a marine isolate of *Aspergillus* sp.. Eur. J. Org. Chem..

[56] Zhu F., Chen G., Chen X., Huang M., Wan X. (2011). Aspergicin, a new antibacterial alkaloid produced by mixed fermentation of two marine-derived mangrove epiphytic fungi. Chem. Nat. Compd..

[57] Bui-Klimke T.R., Wu F. (2015). Ochratoxin A and Human Health Risk: A Review of the Evidence. Crit. Rev. Food Sci. Nutr..

