# Supplementary material for: Bioactive Metabolites from the Endophytic Fungus Aspergillus sp. SPH2
Source: J Fungi (Basel). 2021 Feb 2;7(2):109. doi: 10.3390/jof7020109 (PMC7913058; doi:10.3390/jof7020109)
Supplement: Supplementary file 1 [file jof-07-00109-s001.pdf]

Material and Methods

Insect Bioassay

Spodoptera littoralis, *Myzus persicae* and *Rhopalosiphum padi* colonies were reared on artificial diet [1], bell pepper (*Capsicum annuum*) and barley (*Hordeum vulgare*) plants, respectively. The plants are grown from seeds in pots with commercial substrate. The plants for rearing aphids are infected regularly (bell pepper plants with 4 leaves, barley plants of 10 cm length). The insect colonies and host plants were maintained at 22 ± 1 ºC, >70% relative humidity with a photoperiod of 16:8 h (L:D) in a growth chamber.

The upper surface of *C. anuum* and *H. vulgare* leaf disks or fragments (1.0 cm2) were treated with 10 µl of the test substance. The crude extracts and products were tested at an initial dose of 100 or 50 µg/cm2 respectively. Five Petri dishes (9 cm diam.) or twenty ventilated plastic boxes (2 × 2 cm) with two newly molted *S. littoralis* L6 larvae (< 24 h) or ten apterous aphid adults (24–48 h old) each were allowed to feed at room temperature for *S. littoralis* (< 2 h) or in a growth chamber for the aphids (24 h, environmental conditions as above). Each experiment was repeated 2-3 times (SE < 10%) and terminated when the consumption of the control disks reached 65–75% for *S. littoralis* or after 24 h for aphids. The leaf disk area consumed was measured on their digitalized images (Image J, http://imagej.nih.gov/ij). Settling was measured by counting the number of aphids settled on each leaf fragment. Feeding or settling inhibition (%FI or %SI) was calculated as % FI/%SI = [1 – (T/C) × 100], where T and C are the consumption/settling of treated and control leaf disks, respectively. The antifeedant effects (% FI/SI) were analyzed for significance by the nonparametric Wilcoxon signed-rank test [2].

Nematode Bioassay

A laboratory (ICA-CSIC) root knot nematode (*Meloydogine javanica*) population maintained on tomato (*Lycopersicon esculentum* var. Marmande) plants in pots at 25±1 °C and > 70% relative humidity was used for the experiments. Second-stage juveniles (J2) hatched within 24 h (from egg masses handpicked from infected tomato roots) were used. The experiments were carried out in 96-well microplates (Becton, Dickinson) as described [3]. The organic extracts and pure compounds were tested at initial concentrations of 1.0 and 0.5 mg/mL (final concentration in the well) and diluted serially if needed. The number of dead juveniles was recorded after 72 h. All treatments were replicated four times. The data were determined as percent mortality and corrected according to Scheider-Orelli's (1947) formula.

1. Poitout, S.; Bues, R. Rearing of several species of Lepidoptera Noctuidae on a rich artificial medium and a simplified artificial medium. *Ann. Zool. Ecol. Anim.* **1970**, *2*, 79–91 ⟨hal-02730050⟩.
2. Burgueño-Tapia, E.; Castillo, L.; González-Coloma, A.; Joseph-Nathan, P. Antifeedant and phytotoxic activity of the ses-quiterpene p-benzoquinone perezone and some of its derivatives. *J. Chem. Ecol.* **2008**, *34*, 766–771, doi: 10.1007/s10886-008-9495-2.
3. Andrés, M.F.; Gonzalez-Coloma, A.; Sanz, J.; Burillo, J.; Sainz, P. Nematicidal activity of essential oils: A review. *Phytochem. Rev.* **2012**, *11*, 371–390, doi:10.1007/s11101-012-9263-3.

Table S1. Antifeedant effects of SPH2 extract.

**Extract**	**Concentration (μg/cm²)**	***S. littoralis***	***M. persicae***	***R. padi***
SPH 2	100	45,5 ± 5,6	52,19 ± 7,97	48,8 ± 9,07

Table S2. Nematicidal activity of SPH2 extract.

**Extract**	**Concentration (μg/μL)**	***M. javanica***
SPH 2	20	23.7 ± 3.24
